# Microplasma illumination enhancement of vertically aligned conducting ultrananocrystalline diamond nanorods

**DOI:** 10.1186/1556-276X-7-522

**Published:** 2012-09-25

**Authors:** Kamatchi Jothiramalingam Sankaran, Srinivasu Kunuku, Shiu-Cheng Lou, Joji Kurian, Huang-Chin Chen, Chi-Young Lee, Nyan-Hwa Tai, Keh-Chyang Leou, Chulung Chen, I-Nan Lin

**Affiliations:** 1 Department of Materials Science and Engineering, National Tsing-Hua University, Hsinchu, Taiwan 300, Republic of China; 2 Department of Engineering and System Science, National Tsing-Hua University, Hsinchu, Taiwan 300, Republic of China; 3 Department of Photonics Engineering, Yuan Ze University, Chung-Li, Taiwan 32003, Republic of China; 4 Department of Physics, Tamkang University, Tamsui, Taiwan 251, Republic of China

**Keywords:** Ultrananocrystalline diamond nanorods, Reactive ion etching, Microplasma, Electron field emission properties

## Abstract

Vertically aligned conducting ultrananocrystalline diamond (UNCD) nanorods are fabricated using the reactive ion etching method incorporated with nanodiamond particles as mask. High electrical conductivity of 275 Ω·cm^−1^ is obtained for UNCD nanorods. The microplasma cavities using UNCD nanorods as cathode show enhanced plasma illumination characteristics of low threshold field of 0.21 V/μm with plasma current density of 7.06 mA/cm^2^ at an applied field of 0.35 V/μm. Such superior electrical properties of UNCD nanorods with high aspect ratio potentially make a significant impact on the diamond-based microplasma display technology.

## Background

Microplasma science and technology is an intersection of plasma science, photonics, and materials science, which offers not only a realm of plasma phenomenology but also device functionality [1-4]. Such plasma-based devices exhibit great potential for a broad spectrum of applications in microdisplays, on-chip frequency standards, materials synthesis, elemental analysis, and detectors of environmentally hazardous or toxic gases or vapors [5-11] But due to the insufficient luminous efficiency of the plasma devices [12], development of a cathode material with efficient emission of secondary electrons for improving the initiation efficiency of plasma illumination is thus called for. Among carbon-based materials, diamond is a promising material for applications in various electronic and microelectromechanical devices due to its unparalleled intrinsic properties such as wide energy band gap, chemical inertness, extreme hardness, high thermal conductivity, and negative electron affinity [13-16]. Moreover, diamond materials have large secondary electron emission efficiency which is especially adept for serving as cathode materials in microplasma devices [17]. Recent reports of *n*-type conductivity [18] and high electron field emission (EFE) characteristics in N_2_-incorporated ultrananocrystalline diamond (UNCD) films exhibited the potential of such films for cold cathode emitters [19,20]. These materials are expected to be beneficial for maintaining the plasma excitation when serving as cathode materials for the plasma devices.

It is not the intrinsic properties alone but the surface geometry also which serves in defining the properties for potential applications of materials. In spite of retaining the same chemical composition, nanostructured materials exhibit pronounced variations in the properties in comparison with their bulk and film forms. For instance, it has been demonstrated that diamond coatings on silicon nanostructures significantly reduce the turn-on field (*E*_0_) of EFE [21,22]. Processing of materials to a desired geometry will depend entirely on the intrinsic properties such as hardness, chemical and/or mechanical stability, etc., of the materials. Nanostructures of extremely hard and chemically inert materials such as diamond and other wide-bandgap materials (GaN, Si) have been obtained by the top-down methods including reactive ion etching (RIE) process with or without mask focus ion beam milling and bottom-up approaches [23-33]. Owing to practical applications, it is still of great interest to formulate a low-cost, flexible, and relevant method to fabricate diamond nanostructures with high areal density and high uniformity in a desired geometry.

In this letter, we report the fabrication of vertically aligned UNCD nanorods from *n*-type UNCD films by RIE using nanodiamond (ND) particles as a hard etching mask. We observed that the plasma illumination characteristics of a microplasma cavity were markedly enhanced when the UNCD nanorods were used as the cathode materials, as compared with those using the as-grown UNCD films as cathode. The detailed mechanism of the improvement of the plasma illumination characteristics of the nanorods is investigated.

## Methods

UNCD films were grown on Si substrates in a microwave plasma-enhanced chemical vapor deposition system (2.45 GHz 6″ IPLAS-CYRANNUS, Troisdorf, Germany). Prior to deposition, the substrates were ultrasonicated in methanol solution containing the mixture of ND powders (approximately 5 nm) and titanium powders (approximately 325 nm) for 45 min to facilitate the nucleation. The UNCD films were deposited on substrates using N_2_ (94%)/CH_4_ (6%) plasma with a microwave power of 1,200 W for 1 h. The pressure and the flow rate were maintained at 50 Torr and 100 sccm, respectively. An external heater was used to heat the substrate to a temperature of about 700°C, where the substrate temperature is measured using a thermocouple (*K* type) embedded in the substrate holder. The obtained UNCD films were designated as N_2_-UNCD films. The N_2_-UNCD films were then immersed in a pseudo-stable suspension (ND particles (8 to 10 nm in diameter) and deionized water) and sonicated for 10 min to seed ND particles on the N_2_-UNCD films surface. The ND particle layer on the N_2_-UNCD films is dense, which depends on the suspension quality and time of sonication. After masking, the N_2_-UNCD films were then etched using the RIE process in an O_2_ (80%)/CF_4_ (20%) gas mixture at rf power of 150 W for 30 min. In the process, ND particles acted as etching mask for fabricating vertically aligned N_2_-UNCD nanorods.

The morphologies and microstructures of the samples were examined using field emission scanning electron microscopy (FESEM; JEOL-6500, JEOL Ltd., Tokyo, Japan) and transmission electron microscopy (TEM; JEOL 2100; operated under 200 eV), respectively. The visible Raman (*λ* = 632.8 nm; Lab Raman HR800; Jobin Yvon, Inc., NJ, USA) spectroscopic measurements were performed at room temperature. Hall measurements were carried out in a van der Pauw configuration (ECOPIA HMS 3000, Bridge Technology, USA) to confirm *n*-type conductivity of the films. EFE characteristics of the samples were measured using a molybdenum rod with a diameter of 2 mm as anode, and *IV* characteristics were acquired using Keithley 237 electrometer (Keithley Instruments, Inc., OH, USA). The EFE behavior of the materials was explained using Fowler-Nordheim (F-N) theory [34]. The plasma illumination characteristics of a microcavity, in which an indium tin oxide (ITO)-coated glass was used as anode and the N_2_-UNCD nanorods were used as cathode, were also investigated. The cathode-to-anode separation was fixed by a Teflon spacer (1.0 mm in thickness). A circular hole about 8.0 mm in diameter was cut out from the Teflon spacer to form a microcavity. The plasma was triggered using a pulsed direct current voltage in bipolar pulse mode in Ar environment at a pressure of 100 Torr.

## Results and discussion

FESEM image of the N_2_-UNCD films shows highly dense and uniformly distributed needle-like granular structures in the films (not shown). The root-mean square roughness of the surface is about 7 to 10 nm, and the thickness of the films is about 1 μm. The Hall measurements conducted in the van der Pauw configuration showing the electrical conductivity of the N_2_-UNCD films are found to be 186 Ω·cm^−1^. Vertically aligned N_2_-UNCD nanorods are fabricated by subjecting the N_2_-UNCD films to the RIE process.

**Figure 1:**
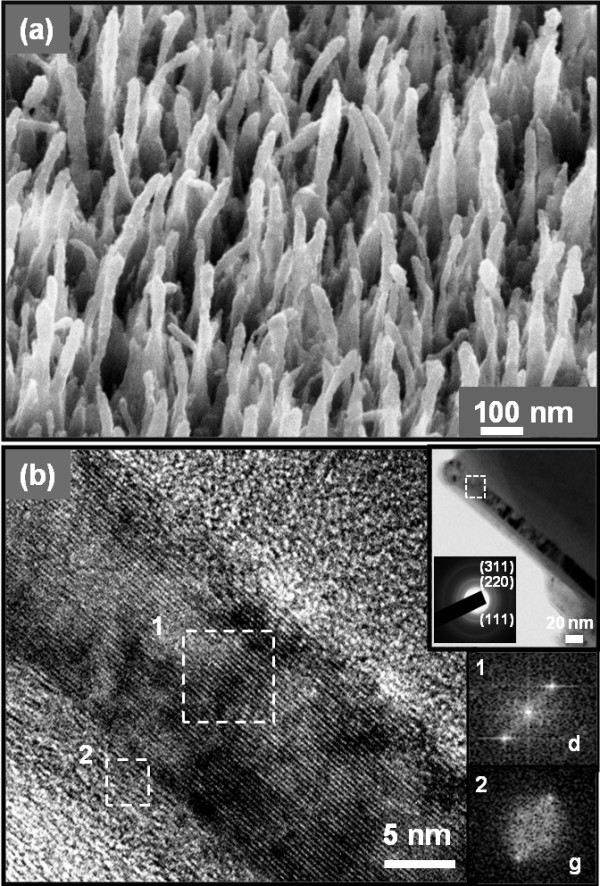
**FESEM morphology and TEM images of N**_**2**_**-UNCD nanorods. **(**a**) FESEM morphology of the N_2_-UNCD nanorods, which are fabricated from N_2_-UNCD films grown using N_2_ (94%)/CH_4_ (6%) plasma at 700°C, using RIE etching process with nanodiamond particles as masking materials. (**b**) High resolution TEM image of N_2_-UNCD nanorod with the upper-right inset showing the low magnification TEM image of N_2_-UNCD nanorod and the corresponding SAED pattern. The insets 1 and 2 show the Fourier-transformed diffractogram (FT images) of the areas marked as 1 and 2, indicating that the UNCD nanorods are encased in a thin shell of graphite content of around 2 to 3 nm thickness. d, diamond; g, graphite.

Figure 1a shows the FESEM image of the vertically aligned N_2_-UNCD nanorods with diameters of about 15 to 20 nm and lengths of about 460 nm. Examination of the films using TEM is necessary to explicitly identify the microstructural nature of the materials. The high resolution TEM image of a single nanorod (Figure 1b) taken from the marked squared region in the low magnification TEM micrograph (upper-right inset of Figure 1b) reveals two crystalline carbon phases, diamond (marked squared region 1 in Figure 1b) and graphite (marked squared region 2 in Figure 1b). The image shows a nanorod with a diameter of about 15 nm, surrounded by graphitic phase. The thickness of the graphitic layer can vary from a few atomic layers to approximately 3 nm. The associated selective area electron diffraction (SAED) pattern of the low-magnification TEM micrograph of the N_2_-UNCD nanorods (upper-right inset of Figure 1b) also clearly shows the presence of two different crystalline phases: a diamond phase (sharp rings designated as d_111_, d_220_, and d_311_) and a graphitic phase (central diffused ring). In addition, the Fourier transformed (FT) diffractogram corresponding to the region 1 of the structure image (FT_1_) clearly illustrates the diamond phase, whereas the FT image corresponding to the region 2 (FT_2_) indicates that these curved parallel fringes correspond to a few layers of graphitic phase. These results confirm that the nanorods are encapsulated by a sheath of graphitic phase. The microstructural studies of N_2_-UNCD films confirmed that this graphitic content is formed during the growth of the films [35]. The presence of abundant CN species in the N_2_/CH_4_ plasma, which was observable in the optical emission spectra (not shown), may preferentially induce the formation of nanorod, along with the graphitic phase encasing the nanorods [36].

**Figure 2:**
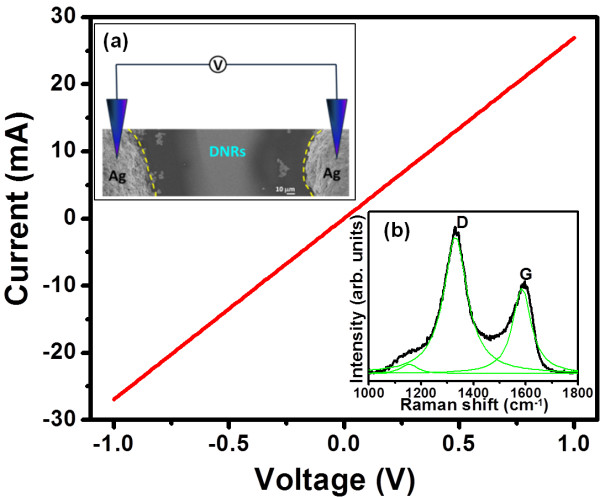
**Current–voltage characteristics and visible-Raman spectrum of N**_**2**_**-UNCD nanorods. **The current–voltage characteristics, which are obtained using Hall measurements with the measuring probes directly in contact with the top of the nanorods (inset **a**), reveal that the N_2_-UNCD nanorods possess good electrical conductivity of 275 Ω·cm − ^1^. Inset (**b**) shows the visible Raman spectrum of the N_2_-UNCD nanorods. d, diamond; g, graphite.

The nanorods are subjected to Hall measurements with the measuring probes directly in contact with the nanorods (inset a of Figure 2), and the electrical conductivity increases to about 275 Ω·cm − ^1^ (Figure 2). The visible Raman spectrum of the N_2_-UNCD nanorods is shown in the inset b of Figure 2. The spectrum is deconvoluted using the multi-peak Lorentzian fitting method. Two prominent resonance peaks are observed in the spectrum. The broadened Raman peak at approximately 1,335 cm^−1^ is attributed to the *D* band, which arises due to disordered carbon, while the peak observed at approximately 1,597 cm^−1^, assigned as the *G* band, is due to the graphitic phase in the nanorods [37], which is in accord with the TEM observation. It must be noted that the absence of a sharp feature at approximately 1,332 cm^−1^ is due to the use of visible Raman spectroscopy, which is more sensitive towards *sp*^2^-bonded carbon. The N_2_-UNCD nanorods exhibit high electrical conductivity due to the increase in the content of *sp*^2^ carbon bonding in the nanorods.

**Figure 3:**
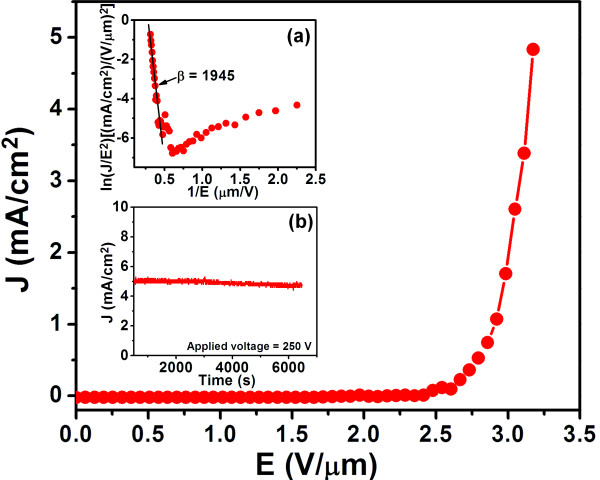
**The electron field emission properties of the N**_**2**_**-UNCD nanorods. **Inset (**a**) shows the corresponding Fowler-Nordheim plot. Inset (**b**) shows the plasma illuminating current stability measurement of a microplasma cavity, which utilized the N_2_-UNCD nanorods as cathode and ITO glass as anode.

The EFE measurements were carried out on the N_2_-UNCD nanorods, and the results are shown in Figure 3 with the inset a showing the F-N plot. An applied field at a current density of 10 μA/cm^2^ was taken as the *E*_0_. The N_2_-UNCD nanorods require only (*E*_0_)_nanorod_ = 2.04 V/μm to turn on the EFE process and reach an EFE current density (*J*_e_)_nanorod_ of 4.84 mA/cm^2^ at an applied field of 3.2 V/μm. Such EFE properties are markedly superior to those of the N_2_-UNCD films with (*E*_0_)_film_ of 4.70 V/μm and (*J*_e_)_film_ of 3.47 mA/cm^2^ at 8.8 V/μm applied field (not shown). The relationships among the current density (*J*_e_), electric field (*E*), work function (*ϕ*), and field enhancement factor (*β*) of an emitter are expressed by the F-N equation: $J_{e}=\left(Ab^{2}E^{2}/\phi \right)\;\text{exp}\;\left(-B\phi^{3/2}/bE\right),\text{where}\;A=1.54\times 10^{-6}{\text{A eV V}}^{-2}$ and $B=6.83\times 10^{9}{\text{eV}}^{-3/2}{\text{V m}}^{-1}$. We fit the high field segments of the F-N curve to the above equation and the results of the fitting are shown as straight segment in the inset a of Figure 3, illustrating that the EFE data fit the F-N model very well. We assume that the *ϕ* value of diamond is assumed as 5.0 eV [38] for estimating the *β* value of N_2_-UNCD nanorods, which is *β*_nanorod_ = 1,945 from the F-N slope. The value of *β*_nanorod_ obtained is larger than that of the N_2_-UNCD films (*β*_film_ = 624), the enhanced value being due to the electrical field at the nanorod tips.

**Figure 4:**
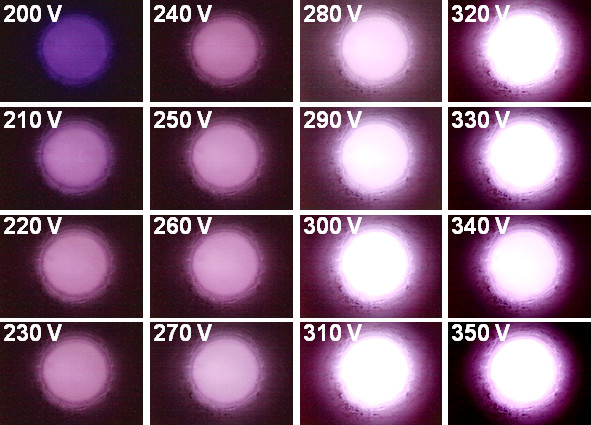
**Plasma illumination characteristics of N**_**2**_**-UNCD nanorods. **The photographs of plasma illumination characteristics of a microplasma cavity which utilized the N_2_-UNCD nanorods as cathode and ITO glass as anode.

Figure 4 shows the series of photographs of the plasma devices at different applied electric fields. These photographs show that the microplasma devices using the N_2_-UNCD nanorods as cathode can be triggered by a voltage of 210 V, and the intensity of the plasma increases monotonously with the applied voltage. The plasma current density of N_2_-UNCD nanorods ((*J*_e_)_nanorod_) also exhibits a similar increase with the increase of applied electric field and reaches 7.06 mA/cm^2^ at an applied field of 0.35 V/μm. The (*J*_e_)_nanorod_ is larger than that of the plasma current density ((*J*_e_)_film_ = 5.30 mA/cm^2^ at an applied field of 0.34 V/μm) of N_2_-UNCD films (not shown). The threshold field (*E*_th_)_nanorod_ for triggering the plasma corresponds to an applied field of (*E*_th_)_nanorod_ = 0.21 V/μm, which is smaller than the *E*_th_ value of N_2_-UNCD film-cathoded microplasma devices (not shown). To evaluate the stability of the plasma illumination from N_2_-UNCD nanorods, the current was monitored over a period of 7,000 s with a constant applied voltage of 250 V (inset b of Figure 3). The plasma (*J*_e_)_nanorod_ of 4.74 mA/cm^2^ is upheld for a period of 6,445 s and shows high life-time stability in comparison with that of the N_2_-UNCD films as well as bare Si. Apparently, the better plasma performance of the microplasma cavity using N_2_-UNCD nanorods as cathode, as compared with that using N_2_-UNCD film as cathode (not mentioning that of the cavity with the bare Si used as cathode) can be ascribed to the superior EFE properties besides the high secondary electron emission efficiency for the N_2_-UNCD nanorod materials.

It should be noted that the electric field required to trigger the Ar plasma is much smaller than the *E*_0_ for inducing the EFE process for both the N_2_-UNCD nanorods and N_2_-UNCD films. The primary reason for such a phenomenon is that the Ar plasma can be triggered whenever the electrons emitted from the cathodes reach a kinetic energy larger than the ionization energy of the Ar species (14.7 eV). Superior EFE properties provide the low ignition threshold for the microplasma easily. After the initiation of the Ar plasma, the cathode materials mainly serve as the source of secondary electrons for maintaining the ignition of the plasma. Better EFE properties of the N_2_-UNCD nanorods no longer show significant superiority in maintaining the plasma in the microcavity.

## Conclusions

In summary, ND particles dispersed on smooth and highly conducting N_2_-UNCD films can be utilized as an etching mask for the fabrication of vertically aligned N_2_-UNCD nanorods. These N_2_-UNCD nanorods show superb plasma illumination characteristics of low threshold field = 0.21 V/μm with high current density of 7.06 mA/cm^2^ at an applied field of 0.35 V/μm. The excellent performance of the N_2_-UNCD nanorods as cathode for the microplasma devices is mainly attributed to the unique granular structure of nanorods and a high proportion of graphitic phase surrounding each nanorod. The utilization of N_2_-UNCD nanorods enhances the illumination performance of the microplasma devices that can be applied to a broad spectrum of applications in microplasma display technologies.

## Competing interests

The authors declare that they have no competing interests.

## Authors’ contributions

KJS and NHT carried out the growth, electrical conductivity, and electron field emission studies of N_2_-UNCD films. SK, JK, and KCL carried out the fabrication of N_2_-UNCD nanorods using RIE process and Raman spectroscopy studies. SCL and CC carried out the microplasma illumination studies. HCC, CYL, and INL carried out the TEM investigation on N_2_-UNCD nanorods. All authors read and approved the final manuscript.

## Authors’ information

KJS is a Ph.D. student of the Department of Materials Science and Engineering, National Tsing-Hua University, Hsinchu, Taiwan. SK is a Ph.D. student of Department of Engineering and System Science of the same university. SCL is a Ph.D. student of the Department of Photonics Engineering, Yuan Ze University, Chung-Li, Taiwan. JK is a post doctoral fellow in the Department of Physics, Tamkang University, Tamsui, Taiwan. HCC is a post doctoral fellow in the Department of Materials Science and Engineering, National Tsing-Hua University, Hsinchu, Taiwan. CYL and NHT are professors in the Department of Materials Science and Engineering of the same university. KCL is a professor in the Department of Engineering and System Science of the same university. CC is a professor in the Department of Photonics Engineering, Yuan Ze University, Chung-Li, Taiwan. INL is a professor in the Department of Physics, Tamkang University, Tamsui, Taiwan.
